# Olive Mill Waste Extracts: Polyphenols Content, Antioxidant, and Antimicrobial Activities

**DOI:** 10.1155/2015/714138

**Published:** 2015-11-29

**Authors:** Inass Leouifoudi, Hicham Harnafi, Abdelmajid Zyad

**Affiliations:** ^1^Laboratory of Biological Engineering, Faculty of Science and Technologies, Sultan Moulay Slimane University, 23 000 Beni-Mellal, Morocco; ^2^Laboratory of Biochemistry, Faculty of Science, Mohamed First University, 60 000 Oujda, Morocco

## Abstract

Natural polyphenols extracts have been usually associated with great bioactive properties. In this work, we investigated* in vitro* antioxidant and antimicrobial potential of the phenolic olive mill wastewater extracts (OWWE) and the olive cake extracts (OCE). Using the Folin Ciocalteux method, OWWE contained higher total phenol content compared to OCE (8.90 ± 0.728 g/L versus 0.95 ± 0.017 mg/g). The phenolic compounds identification was carried out with a performance liquid chromatograph coupled to tandem mass spectrometry equipment (HPLC-ESI-MS). With this method, a list of polyphenols from OWWE and OCE was obtained. The antioxidant activity was measured in aqueous (DPPH) and emulsion (BCBT) systems. Using the DPPH assay, the results show that OWWE was more active than OCE and interestingly the extracts originating from mountainous areas were more active than those produced from plain areas (EC_50_ = 12.1 ± 5.6 *μ*g/mL; EC_50_ = 157.7 ± 34.9 *μ*g/mL, resp.). However, when the antioxidant activity was reversed in the BCBT, OCE produced from plain area was more potent than mountainous OCE. Testing by the gel diffusion assay, all the tested extracts have showed significant spectrum antibacterial activity against* Staphylococcus aureus*, whereas the biophenols extracts showed more limited activity against* Escherichia coli* and* Streptococcus faecalis*.

## 1. Introduction

In the recent years, the interest of natural antioxidants, particularly polyphenols, in relation to their therapeutic and health beneficial properties has significantly increased. Indeed, polyphenols are known for decades for their antioxidant activity [1], which was then confirmed by more recent studies [2, 3]. Mediterranean olive mill wastes are rich on these active ingredients and antioxidant activity of olive oil mill waste phenolic extracts had already been tested [4, 5]. These* in vitro* tests have usually shown an inhibitor effect of oxidation reactions and have attracted increasing attention as potential agents for preventing and treating many oxidative stress-related diseases. One of the first works, which has used olive mill waste as a potential source of natural antioxidants, was published in 1988 [6]. The current work evaluates the phenolic content of olive byproducts and its bioactivities. It may be considered as one of the rarely investigations of antioxidant activity of Moroccan olive mill wastes witch is distinguished from the most Mediterranean olive mill wastes by the nature of the bioclimatic conditions.

Furthermore, the antimicrobial activity was identified in the early twentieth century but has been rarely explored [7]. Most studies of antimicrobial activity have focused on ecological and environmental consequences [8] or on agronomic applications [9]. Antimicrobial activity of olive mill waste extracts was early recognized and linked to the biophenols content [10]. However, the antimicrobial activity of olive cake and olive wastewater phenolic extracts and pure biophenols has been rarely tested against human pathogens [11, 12].

Moreover, beyond demonstrating the antioxidant and antimicrobial activity of olive byproducts polyphenol extracts, a few studies have an interest in comparing both the antioxidant and the antimicrobial effects of phenolic OCE to OWWE extracts, much less evaluating these effects in relation to the bioclimatic collection areas, from which they are originating. Therefore, this work aims to study the* in vitro* antioxidant and antibacterial potentials of Moroccan olive mill waste extracts and the relationship with their phenolic composition.

## 2. Materials and Methods

### 2.1. Chemical Reagents

All solvents and chemicals were obtained from Sigma Chemical Co., Saint Quentin (France). Bacteria strains were originally obtained from the laboratory of Biological Engineering, Faculty of Science and Technology, Sultan Moulay Slimane University, Beni-Mellal, Morocco.

### 2.2. Plant Material

*Moroccan Picholine* olives variety was identified and authenticated by Pr. A. Boulli, Department of life sciences, Sultan Moulay Slimane University, and stored as a voucher specimen in the Faculty of Science and Technologies, Beni-Mellal, Morocco. Samples of olive cake (solid waste) and olive wastewaters (liquid waste) were collected in mills from two areas of Tadla-Azilal region in Morocco, plain and mountainous areas, during the winter of 2012. These samples were produced from the three-phase centrifugation oil extraction process of red-black olives maturation stage.

### 2.3. Phenolic Compounds Extraction

#### 2.3.1. Olive Cake Samples

Dry olive cake samples (60 g each) were grounded, sifted, and then defatted with 500 mL of hexane in a soxhlet apparatus for four hours. Defatted olive cake samples were subjected to soxhlet extractions using ethanol solvent. Olive cake samples (60 g) were placed in extraction thimbles into the soxhlet apparatus. 500 mL of ethanol was placed in a round flask (500 mL capacity) and then the flask was connected to the soxhlet extractor for 12 h at 70°C of continuous extraction [13]. The resulting olive cake extracts (OCE) were concentrated by rotary evaporator and freeze stored at −18°C for further analysis.

#### 2.3.2. Olive Wastewater Samples

Olive mill wastewater was defatted with hexane (1 : 1, (v/v)) and then clarified by centrifugation (4000 rpm, 15 min). Phenolic compounds in defatted and clarified olive mill wastewaters were twice extracted by the liquid-liquid extraction method using ethyl acetate (1 : 1, v/v) and 4000 rpm, 10 min centrifugation. The ethyl acetate phase was evaporated and the residue was stored at −18°C for subsequent analysis.

### 2.4. Total Phenolic Compounds Content (Spectrometric Measurement)

The total phenolic compounds content in each extract was determined by spectrophotometry using the Folin-Ciocalteu method [31, 32] with some modifications. Briefly, 2.5 mL portion of Folin-Ciocalteu reagent 0.2 N was mixed with 0.5 mL of the sample. The reaction was kept in the dark for 5 min. Then, 2 mL of a sodium carbonate solution (75 g/L) was added to the mixture and the reaction was kept in the dark for 1 h. the absorbance was measured at 760 nm and 765 nm for OCE and OWWE, respectively. Results were expressed as gallic acid equivalents (GAE).

### 2.5. HPLC/ESI-MS Analysis

High-performance liquid chromatography-mass spectrometry analysis was performed at 279 nm and 30°C using a RP C18 column (150 × 4.6) × 5 *μ*m with a Thermo Fisher apparatus equipped with a Surveyor quaternary pump coupled at a PDA detector (diode array detector: 200–600 nm) and an LCQ Advantage (ESI) ion trap mass spectrometer (Thermo Finnigan, San Jose, CA). The injected volume was 20 *μ*L. The mobile phase (0.5 mL/min) consisted of solvent A: TFA 0.05% in water and solvent B: TFA 0.05% in ACN. A Six-step gradient was applied, for a total run time of 76 min, as follows: starting from 80% solvent A and 20% solvent B increasing to 30% solvent B over 30 min, then isocratic elution for 10 min, increased to 30% solvent B over 10 min, to 40% over 30 min, and to 20% solvent B over 2 min, and finally isocratic elution for 4 min. ESI ionization conditions were spray voltage 4 KV, capillary 350°C, 14 V. Pure nitrogen was the sheath gas and pure helium was the collusion gas. The full scan mass data *m*/*z* was obtained in positive mode and ranged from 100 to 2000 Da.

### 2.6. Antioxidant Activity

#### 2.6.1. Free Radical Scavenging Activity Measurement (DPPH Method) [26]

The DPPH (2,2-diphenyl-1-picrylhydrazyl) assay was carried out in a 96-well microtiter plate. The samples and positive control, Vitamin C, were diluted with methanol to prepare sample concentrations equivalent to 200, 100, 50, 25, 12.5, 6.25, and 3.125 *μ*g of dried sample/mL solutions. 150 *μ*L of 0.004% DPPH solution was pipetted into each well of 96-well plate followed by 8 *μ*L of the sample solutions. The plates were incubated at 37°C for 30 min and the absorbance was measured at 540 nm, using ELISA microtiter plate reader. The experiment was performed in triplicate and % scavenging activity was calculated using the following equation:

(1) $$\begin{array}{c} \%\text{ Scavenging}=\left(\;A_{o}-\frac{A_{s}}{A_{o}}\;\right)*100, \end{array}$$

where *A*_*o*_ is the absorbance of the control and *A*_*s*_ is the absorbance of the sample at 540 nm.

#### 2.6.2. Antioxidant Activity Measurement Using *β*-Carotene Bleaching Test (BCBT) [33]

In this assay, linoleic acid (2 mg) was added to Tween 40 (200 mg) and *β*-carotene solution (2 mg in 1 mL chloroform) in a round bottom flask. The chloroform was evaporated completely by heating at 37°C under vacuum for 10 min. Aerated water (100 mL) was added in portions with vigorous shaking. 2 mL of this reaction was transferred to test tubes and 0,5 mL of the tested samples prepared at different concentrations (10 *μ*g/mL, 25 *μ*g/mL, 50 *μ*g/mL, 100 *μ*g/mL, and 200 *μ*g/mL) was added. Absorbance at 490 nm of the control (linoleic acid ID ß-carotene) was measured immediately and time was assigned as *T*_0_. The absorbance was remeasured after 24 h incubation at room temperature (*T*_24_). Values are presented as means ± SD of three parallel measurements.

The percentage inhibition of ß-carotene bleaching was calculated using the following formula:

(2) $$\begin{array}{c} \%\text{ Inhibition}=\left[\;\frac{\left(A_{24}-C_{24}\right)}{\left(C_{0}-C_{24}\right)}\;\right]*100, \end{array}$$

where *A*_24_ is the absorbance of the test extract at *T*_24_, *C*_24_ is absorbance of the control at *T*_24_, and *C*_0_ is the absorbance of the control at *T*_0_.

### 2.7. Antimicrobial Activity [34]

Antimicrobial activity was tested against three microorganisms:* Staphylococcus aureus* and* Streptococcus faecalis*, both Gram-positive bacteria, and* Escherichia coli* as Gram-negative bacteria. Bacteria were cultured in a Mueller Hinton agar medium for 12 h at 37°C. The disc diffusion method was used to determine the antimicrobial activities of OCE and OWWE diluted in DMSO so as to test concentrations of 1.5, 3, and 6 mg/disc for OCE and 1, 2, and 4 mg/disc for OWWE. Agar plates (4 mL/plate) were prepared, allowed to set, and surface dried at 25°C for 15 min. Bacterial cultures were incubated at 37°C for 24 h in order to have a microbial suspension having turbidity nearest 10^5^–10^6^ CFU/mL. 100 *μ*L of the inoculum (3 × 10^6^ CFU/mL) was spread plated on nutrient agar plates. Blank sensitivity discs, 6 mm, were allowed to warm to room temperature for 1 h and then impregnated with 25 *μ*L of each extract or controls and then left to dry in a sterile Petri dish for 90 min. Negative controls for standards and extracts were 25%, 50%, and 100% DMSO. Positive controls were amoxicillin discs (25 *μ*g), chloramphenicol (30 *μ*g), and ceftriaxone (30 *μ*g). The plates were then incubated for 24 h at 37°C. The diameter of the inhibition zone was measured in mm (including disc) with calipers; three replicates were performed and the assays were duplicated.

## 3. Results

### 3.1. Phenolic Compounds Content

The characterization of the biophenols content of OWWE and OCE is provided in Table 1. OWWE has the higher amounts of total phenols compared to OCE, as measured by Folin Ciocalteu assay. The OWWE phenolic content was about 10 times more than OCE phenolic content (Table 1). The levels of mountainous biophenols extracts were interestingly higher than plain biophenols extracts. This difference in total phenolic content can be explained by the impact of geographic and climatic conditions on the determination of polyphenols content in plants [23, 35].

### 3.2. Phenolic Compounds Identification

**Figure 1:**
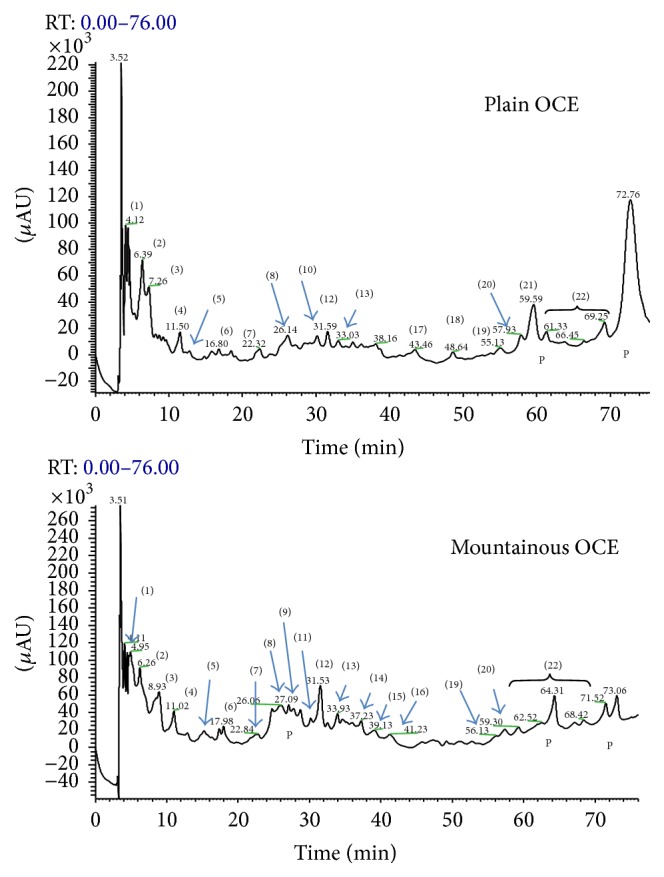
HPLC chromatograms of the phenolic profile of OCE. Peaks identities: (1) hydroxytyrosol glucoside, (2) hydroxytyrosol, (3) tyrosol, (4) vanillic acid, (5) sinapic acid, (6) syringic acid, (7) caffeic acid, (8) elenolic acid, (9) oleuropein aglycone, (10) verbascoside, (11) rutin, (12) luteolin, (13) quercetin, (14) luteolin-7-rutinoside, (15) luteolin-7-glucoside, (16) apigenin, (17) methoxyluteolin, (18) naringenin (19) ligstroside aglycon, (20) ligstroside, (21) oleuropein, (22) secoiridoids derivatives and (P) polymeric substances.

**Figure 2:**
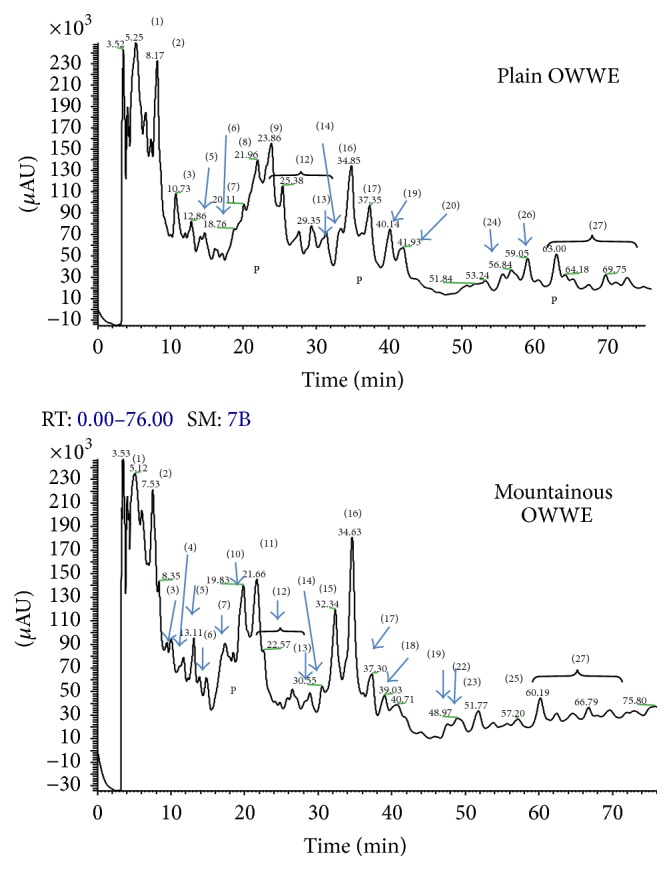
HPLC chromatogram of the phenolic profile of OWWE. Peaks identities: (1) hydroxytyrosol glucoside, (2) hydroxytyrosol, (3) tyrosol, (4) vanillic acid (5) sinapic acid, (6) syringic acid, (7) caffeic acid, (8) p-coumaric acid, (9) dihydroxymandelic acid, (10) vanillin, (11) 3,4,5 trimethoxybenzoic acid, (12) secoiridoids derivatives, (13) verbascoside, (14) rutin, (15) luteolin-7-rutinoside, (16) luteolin-7-glucoside, (17) luteolin, (18) apigenin, (19) nüzhenide, (20) quercetin, (21) apigenin-7-rutinoside, (22) apigenin-7-glucoside, (23) oleuropein, (24) oleuropein aglycon (25) ligstroside, (26) ligstroside aglycon, (27) secoiridoids derivatives and (P) polymeric substances.

HPLC provided separation of individual biophenols in the OCE and OWWE as illustrated in Figures 1 and 2, respectively, for detection at 279 nm, where both qualitative and quantitative differences between mountainous and plain areas are observed. Identification of biophenols was performed by comparing retention times of standards in HPLC-ESI and confirmed by relevant molecular mass data from LC–MS. The major individual biophenols identified in the OCE and OWWE were particularly characterized at five classes, namely, simple phenols, phenolic acids, derivatives secoiridoids, flavonoids, and lignans (Tables 2 and 3). Furthermore, the phenolic composition seems to be related to the impact of bioclimatic conditions.

As the main aim of this study was to screen olive mill waste extracts for biological activities, a detailed characterization of individual compounds was not attempted and only the major peaks appearing at 279 nm were identified to assist in understanding the relation between the chemical composition and the observed bioactivities.

### 3.3. Antioxidant Activity

#### 3.3.1. DPPH Assay

**Figure 3:**
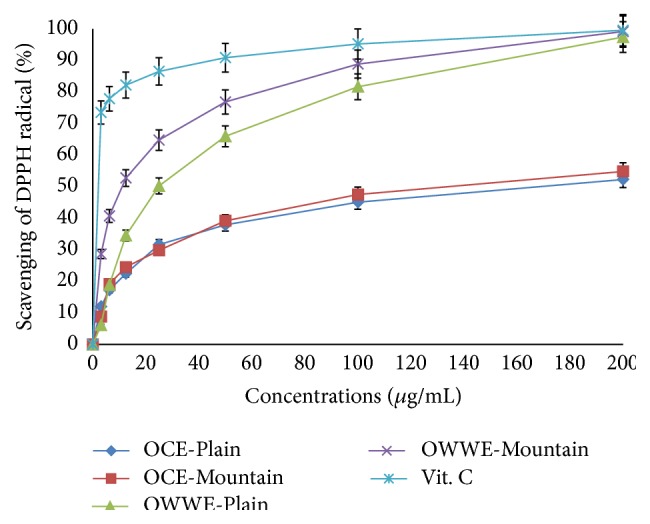
Kinetics of DPPH radical scavenging activity of OCE and OWWE.

Both OCE and OWWE showed concentration-dependent DPPH radical scavenging activity with a high correlation at concentrations less than 200 *μ*g/mL (OWWE-Plain; *R* ^2^ = 0.869, OWWE-Mountain *R* ^2^ = 0.952, OCE-Plain; *R* ^2^ = 0.722, OCE- Mountain; *R* ^2^ = 0.883, Vitamin C; *R* ^2^ = 0.998) (Figure 3). EC_50_ is inversely proportional to antioxidant activity and hence OWWE was more active than OCE in trapping DPPH radicals (Table 4).

Antiradical activity EC_50_ (*μ*g/mL) was defined as the concentration of extracts necessary to decrease the initial DPPH radical concentration by 50%. Values are means standard deviation (SD) of three measurements (*P* < 0.05%).

The difference in activity decreased gradually upon increasing the dose; at EC_50_, OWWE was 13 times more active than OCE in comparison to the mountainous area extracts (EC_50_ = 12.1 ± 5.6; EC_50_ = 157.7 ± 34.9 *μ*g/mL, resp.) while it was only 5 times more active for plain area extracts (EC_50_ = 30.7 ± 4.4; EC_50_ = 168.0 ± 48 *μ*g/mL) (Table 4). This result may be attributed to the highest concentrations of antioxidant phenolic compounds and the nature of the individual phenolic compounds present in the OCE and OWWE extracts. However, for the positive control, EC_50_ value was 3.2 ± 0.6 *μ*g/mL.

#### 3.3.2. BCBT Assay

**Figure 4:**
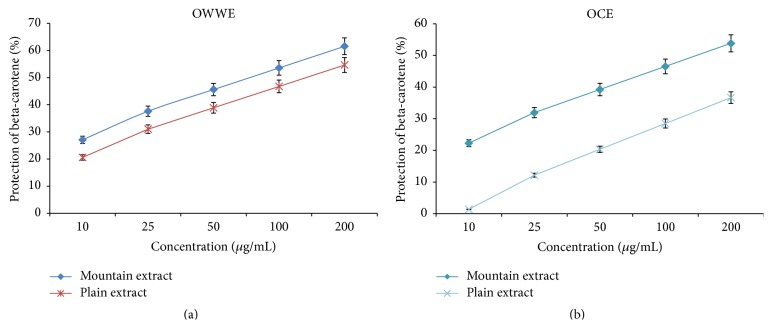
Dose-response curve of antioxidant activity of OCE and OWWE in BCBT.

Both OCE and OWWE protected linoleic acid and hence minimize decolorization of ß-carotene in the BCBT test (Figure 4). OWWE, particularly that originating from mountainous area, showed the higher capacity for oxidation's inhibition with an EC_50_ = 81.3 ± 1.2 *μ*g/mL compared to that originating from plain area (EC_50_ = 131.8 ± 10.3 *μ*g/mL). However, OCE have shown a lower antioxidant activity, and conversely of the results of the DPPH assay, mountainous extracts have shown a very low antioxidant activity in the BCBT test (less than 50% of oxidation's inhibition for the highest concentration: 200 *μ*g/mL) compared to the plain extract (EC_50_ = 139.1 ± 4.56 *μ*g/mL).

### 3.4. Antimicrobial Activity

No antimicrobial activity was observed for the negative control (DMSO) at the tested concentration, while positive controls were active against the studied bacteria except amoxicillin (25 *μ*g) which did not show any antibacterial activity against* Escherichia coli *and* Staphylococcus aureus* [36, 37] (Table 5).

Excepting OWWE mountainous extract (5 mg), no significant antibacterial activity of the tested extracts was observed against* Escherichia coli *and* Streptococcus faecalis*. However,* Staphylococcus aureus* was sensitive to the major tested extracts in a dose-dependent manner. At lower concentrations, the extracts showed various antibacterial effects, but at 5 mg/disc all the samples were active against this strain with similar inhibition zones to those of the positive controls (chloramphenicol 14.2 ± 0.5 mm; ceftriaxone: 15.6 ± 0.4 mm).* Streptococcus faecalis *and* Escherichia coli *seem to be resistant to even high concentrations. No significant differences in activity were observed between OCE and OWWE phenolic extracts whatever their geographical origin (plain or mountain).

## 4. Discussion

### 4.1. Phenolic Composition Extracts

The difference of OCE and OWWE phenolic composition may be attributed to several parameters. It can be according to the olive variety, climate conditions, cultivation practices, the olive storage time, and the olive oil extraction process [22, 29]. Olive mill waste samples were chosen, as the purpose of this study was not to assess differences due to olive variety and olive oil extraction process. Indeed, the total content of phenolic compounds in our extracts appears to be interestingly correlated with the bioclimatic origin and climate conditions (Table 1) but varietal differences cannot be ignored. In this context, other studies have been clearly demonstrated the impact of geographical and climatic conditions on the determination of polyphenols content in plants [23, 28, 35]. Moreover, olive mill waste's composition was studied in various recent studies [17, 35]. It was characterized by its complexity and it was found being rich in hydroxytyrosol and secoiridoids derivatives [3, 14, 28]. HPLC with detection by ESI-MS provides valuable information on phenolic composition. The individual biophenols identified in the OCE and OWWE were classified at five classes, namely, simple phenols, phenolic acids, derivatives secoiridoids, flavonoids, and lignans (Tables 2 and 3). These results were consistent with those found by Suárez et al. [19] and Ramos et al. [17]. Qualitative and quantitative differences are obvious between the profiles of OWWE and OCE (Figures 1 and 2). OWWE had the higher total phenol content consistent with its greater abundance of individual phenols with the exception of oleuropein, ligstroside, and verbascoside, which were higher in OCE. Our results show that hydroxytyrosol is the major compound identified in Moroccan olive cake and olive wastewater phenolic extracts. It has been identified and characterized in the olive cake and olive mill wastewater and had been demonstrated as a major antioxidant agent [5, 17]. Tyrosol was also detected in both extracts at an important level. This phenol was particularly characterized by its important antioxidant effect [14]. The reduced levels of oleuropein, ligstroside, and verbascoside can be attributed to hydrolysis of hydroxytyrosol, tyrosol, and hydroxytyrosol glucoside. Oleuropein and verbascoside have been identified in various studies for their important antioxidant and antimicrobial potential [4, 17]. The amount of flavonoids recovered from OWWE was notably larger than OCE. Moreover, apigenin-7-glucoside and apigenin-7-rutinoside, which could not be detected in OCE, were identified in OWWE chromatograms (Figure 2). Other secoiridoids derivatives were detected as well as those of OCE and OWWE with an observed abundance in OWWE especially those from mountainous areas. The most answered were 3,4-DHPEA-EA, 3,4-DHPEA-EDA, and hydroxytyrosol glucoside [4, 5, 19]. The elenolic acid, the main fragment of the oleuropein degradation, was mostly found in OCE. It can be considered as an important antimicrobial and antiviral agent [22].

### 4.2. Antioxidant Activity

The determination of the antioxidant activity of plant extracts requires a multidimensional evaluation of antioxidant activities combined with special tests. Thus, the free radical scavenging DPPH and the bleaching test BCBT were chosen for this study. The results differ depending on the test used. This can be explained by the sensitivity of each test to the analyzed extracts. With the DPPH method, both extracts tested have been active for trapping free radicals DPPH according to the phenolic extract dose (Figure 3). The EC_50_ values, inversely proportional to the antiradical scavenging DPPH showed that OWWE was more active than OCE and interestingly, the mountainous phenolic extract were more active than the plain phenolic extracts (Table 4). The results confirmed the existence of a good correlation between the antioxidant potential and the total polyphenol content [17, 38]. OCE and OWWE showed a linear correlation coefficient: *R* ^2^ = 0.954 and *R* ^2^ = 0.977, respectively, indicating that 95% and 97% of the antioxidant capacity were due to the contribution of the phenolic compounds and they represent the dominant antioxidants in these extracts [5, 39]. In contrast to the DPPH assay, OCE mountainous extract was more efficient to protect elenolic acid than OCE plain extract in BCBT assay, which can be attributed to the high specificity of the BCBT assay for lipophilic compounds. This suggests the more hydrophobic nature of antioxidants present in OCE originating from plain area compared to those originating from mountainous area. This result could be attributed to the hydrolysis of some compounds present in olive mill waste extracts such as verbascoside [14]. Interestingly, hydroxytyrosol glucoside and caffeic acid, the major constituent compounds of verbascoside, were detected at significant level in OCE plain extract with reduced level of verbascoside concentration. The observed antioxidant activity can also be related to the chemical composition of the evaluated extracts, which were rich in hydroxytyrosol, secoiridoids and derivatives, phenolic acids, and flavonoids. With reference to the phenolic compounds chemical structures, hydroxytyrosol, which usually proved high radical scavenging activity, has a 3,4 dihydroxyl structure bonded to an aromatic ring. This gives it a greater activity compared to tyrosol, for example, which has a similar structure, but with only one hydroxyl group bound to an aromatic ring [17]. These data suggest the importance of the hydroxylation of the aromatic ring of the compounds compared to the phenolic compounds with a single hydroxyl group. The extracts with the highest levels of secoiridoids and derivatives mainly oleuropein, verbascoside, hydroxytyrosol glucoside, and oleuropein aglycon showed also a significant antiradical potential [5, 14]. Furthermore, Fki, et al. [40] measured the antiradical activity of phenolic acids and have demonstrated a high antioxidant activity of caffeic acid. However, the compounds with one hydroxyl group such as p-coumaric acid and syringic acid have showed low antioxidant activity. Moreover, flavonoids, especially luteolin, luteolin-7-glucoside, quercetin, and rutin, were identified among the major phenolic compounds of the olive mill waste. Their characteristic structure with three aromatic rings gives them an important antioxidant activity specifically due to the presence of 3 to 5 hydroxyl groups [5]. However, further purification and fractionation are required to identify the potent antioxidant from the individual active compounds of these naturel extracts.

### 4.3. Antimicrobial Activity

In comparison to the antibiotic antibacterial activity, none of the extracts exhibit a significant activity against* Escherichia coli* and* Streptococcus faecalis* except OWWE mountainous extract at higher concentration (5 mg/mL) against* Streptococcus faecalis* (Table 5). In contrast,* Staphylococcus aureus* was susceptible to the major tested phenolic extracts. Most plant extracts show activity against Gram-positive bacteria but activity against Gram-negative bacteria is a critical measure of success [14]. Yangui et al. [41] demonstrated that hydroxytyrosol, tyrosol, and luteolin showed a good antimicrobial activity against Gram-positive bacteria. Indeed, Figures 1 and 2 showed that hydroxytyrosol and luteolin were among the major constituents of both extracts with a higher content in mountainous OWWE. This was consistent with the significant antibacterial activity of OWWE against* Staphylococcus aureus* and* Streptococcus faecalis* at higher concentration. Other studies showed that flavonoids in particular quercetin [42] and luteolin [43] could be considered as important antibacterial compounds especially against Gram-positive bacteria. Moreover, various antimicrobial activities were mainly attributed to phenolic acid especially caffeic acid, vanillic acid, p-coumaric acid, and 4-hydroxybenzoic acid [12, 44], verbascoside [45], and oleuropein and hydroxytyrosol [14]. Furthermore, no antibacterial activity was observed against* Escherichia coli*. This bacterial strain seems to be very resistant to both of olive mill waste extracts. The different activities against Gram-negative and Gram-positive bacteria may be rationalized by considering differences in cell wall composition. Gram-negative bacteria have a lipopolysaccharide component in their outer membrane that makes them more resistant to antibacterial compounds. OMW biophenols are essentially hydrophilic; the more lipophilic constituents are partitioned into the olive oil during processing. Furthermore, no correlation has been observed between antimicrobial activity and the polyphenol content (*R* ^2^ = 0.00). Similar results were obtained by Pérez et al. [46] in the evaluation of antibacterial activity of olive mill wastewater extracts. Confirming these data, there is no standard method evaluation criteria for the detection of antimicrobial activity in plant extracts [47]. Differences in bacterial strains, growth media and the inoculum size, make comparison of antimicrobial data of plant extracts from different sources very difficult. Other similar works suggested differences in used methods and the relative purity of the extract used in the tests [48]. Nevertheless, some studies showed no selective antimicrobial activity against both Gram-positive and Gram-negative bacteria [49].

## 5. Conclusion

In addition to the extraction and identification of OMW biophenols, OCE and OWWE phenolic extracts in the present study gave promising results in antioxidant and antibacterial activities. It was demonstrated that OWWE, especially those from mountainous areas, were rich on biophenols compounds and more active than those of OCE in the inhibition of the oxidation reactions. This activity has been suggested to be related to the phenolic content amount and to the nature of the phenolic composition extracts. Thus, it was concluded that OWWE was the most promising antioxidant source to contribute to further potential biological properties in the biomedical domains especially as natural anticancer agents. Indeed, studies in cells cancer molecular biology of these extracts will be considered in our further research works. Furthermore, the studied extracts were effective in inhibiting the growth of* Staphylococcus aureus* indicating that such extracts may present an antimicrobial activity against Gram-positive bacteria.

## Figures and Tables

**Table 1:** Total phenolic content in OCE and OWWE.

Area	Total phenolic content	
	OCE (mg GAE^*∗*^/g)	OWWE (g GAE/L)
Mountain	0.950 ± 0.017^a^	8.90 ± 0.728^c^
Plain	0.551 ± 0.027^b^	5.17 ± 0.057^d^

Values are means of duplicate analysis and expressed as gallic acid equivalent.

Different letters mean significant differences ± standard deviation (*P* < 0.05) (Student's test).

^*∗*^GAE: gallic acid equivalent.

**Table 2:** Major phenolic compounds identified in OCE.

Compounds	[M-H]^+^	Main fragments	Area		References^b^
	(*m*/*z*)^a^	ESI-MS	P	M	
*Phenolic alcohols*					
Tyrosol	139		ID	ID	[5, 14–19]
Hydroxytyrosol	155		ID	ID	[5, 14–16, 20–22]
*Phenolic acids*					
Vanillic acid	169		ID	ID	[15, 17, 19, 22–24]
Caffeic acid	181		ID	NI	[4, 5, 13, 14, 16, 19, 23]
Sinapic acid	225		ID	ID	[22, 23]
Dihydroxymandelic acid	185		ID	ID	[15]
Vanillin	153		ID	ID	[5, 16, 23, 24]
*Secoiridoids and derivatives*					
Oleuropein	541	227/225, 303/301	ID	ID	[4, 5, 14, 15, 17, 24, 25]
3,4-DHPEA-EA^b^	379		ID	ID	[5, 16, 17, 19, 20]
3,4-DHPEA-EDA^b^	321		ID	NI	[5, 17]
Oleuropein derivatives	369	225/223, 141/139	NI	ID	[5, 17]
Elenolic acid (p-HPEA-EDA)	243	225/223, 197/195, 179/177	ID	ID	[5, 15–17, 19, 22]
Ligstroside	525	395/393	ID	ID	[5, 14, 15, 23]
p-DHPA-EA^b^	363		NI	ID	[5, 16, 17, 19, 20]
Ligstroside derivatives	337	217/215, 155/153	ID	ID	[5, 20, 24]
Ligstroside derivatives	293		ID	NI	[20]
Ligstroside derivatives	395	259/257	ID	ID	[20]
Hydroxytyrosol glucoside	317	137/135	ID	NI	[5, 15, 16]
Oleoside	391		ID	NI	[14, 15, 23, 26]
Verbascoside	365		ID	NI	[14, 15, 23, 24]
*Flavonoids*					
Apigenin	271		NI	ID	[5, 14, 16, 19, 22, 23]
Luteolin	287	153/151	ID	ID	[5, 14, 15, 20, 22, 24]
Luteolin-7-glucoside	449	287/285	NI	ID	[5, 14–16, 19, 23, 24]
Nüzhenide	685		NI	ID	[19]
Quercetin	303		ID	ID	[14, 22, 23]

^a^Masse/charge, in the positive mode.

ID: Identified; NI: not identified.

P: plain/M: mountain.

^b^3,4-DHPEA-EA: oleuropein aglycon, p-DHPA-EA: ligstroside aglycon, 3,4-DHPEA-EDA: oleuropein aglycon isomer in aldehyde form, and 3,4-DHPEA-AC: hydroxytyrosol acetate.

**Table 3:** Major phenolic compounds identified in OWWE.

Compounds	[M-H]^−^	Main fragments	Areas		References
	(*m*/*z*)^a^	ESI-MS^b^	P	M	
*Phenolic alcohols*					
Tyrosol	137		ID	ID	[3, 14, 27, 28]
Hydroxytyrosol	153		ID	ID	[3, 18, 27–29]
*Phenolic acids*					
Vanillic acid	167		NI	ID	[18, 24]
Sinapic acid	223		ID	NI	[22, 23]
Syringic acid	197		ID	ID	[3, 22, 23, 30]
Caffeoylquinic acid	353	191	ID	NI	[24]
3,4,5 Trimethoxybenzoic acid	211		NI	ID	[30]
Vanillin	151		NI	ID	[23, 24, 27]
*Secoiridoids and derivatives*					
3,4-DHPEA-EDA^b^	319	227, 183	ID	NI	[28]
ME 3,4 DHPEA-EA^b^	409		ID	NI	[5]
Oleuropein derivatives	365	214, 307	NI	ID	[5]
Ligstroside	523	335, 259	NI	ID	[20, 23]
p-DHPA-EA^b^	361		ID	NI	[28]
Ligstroside derivatives	337	155	ID	NI	[20]
Ligstroside derivatives	393	257, 137	ID	NI	[20]
Elenolic acid	241		NI	ID	[15]
3,4-DHPEA-AC^b^	195		ID	NI	[15]
Hydroxytyrosol glucoside	315	150	ID	ID	[17]
Oleoside	389	209	ID	NI	[17]
Verbascoside	623	526, 277	ID	ID	[14, 18, 24, 28]
*Flavonoids*					
Apigenin-7-rutinoside	577		NI	ID	[22, 23]
Apigenin-7-glucoside	477		NI	ID	[22, 23]
Luteolin	285		ID	ID	[20, 22, 23, 28]
Luteolin-7-glucoside	447		ID	ID	[18, 23, 24, 28]
Luteolin-7-rutinoside	593		NI	ID	[22–24]
Nüzhenide	685		ID	NI	[22, 23]
Rutin	609		ID	ID	[18, 23, 24]
*Lignans*					
1 Acetoxypinoresinol	415		ID	ID	[22]
Pinoresinol	357		NI	ID	[22]

^a^Masse/charge, in the negative mode.

ID: identified; NI: not identified.

P: plain/M: mountain.

^b^3,4-DHPEA-EA: oleuropein aglycon, p-DHPA-EA: ligstroside aglycon, 3,4-DHPEA-EDA: oleuropein aglycon isomer in aldehyde form, ME 3,4 DHPEA-EA: oleuropein aglycon in methyl form, and 3,4-DHPEA-AC: hydroxytyrosol acetate.

**Table 4:** Scavenging effects (EC_50_ *μ*g/mL) of OCE and OWWE on DPPH free radicals.

	OCE		OWWE		Vitamin C
	Plain area	Mountainous area	Plain area	Mountainous area	
EC_50_ (*μ*g/mL)	168.0 ± 48	157.7 ± 34.9	32.7 ± 4.5	12.1 ± 5.6	3.2 ± 0.6

Antiradical activity EC_50_ (*μ*g/mL) was defined as the concentration of extracts necessary to decrease the initial DPPH radical concentration by 50%. Values are means standard deviation (SD) of three measurements (*P* < 0.05%).

**Table 5:** Antimicrobial activity of OCE and OWWE.

Test substance (dose/disc)	Inhibition zone (mm)		
	Bacteria		
	*Escherichia coli*	*Staphylococcus aureus*	*Streptococcus faecalis*
*OCE*			
Plain extract (1.25 mg)	0	13,2 ± 0,4	0
Plain extract (2.50 mg)	0	14,6 ± 0,1	0
Plain extract (5 mg)	11,65 ± 0,75	15,7 ± 0,7	11,1 ± 0,1
Mountainous extract (1.25 mg)	0	0	0
Mountainous extract (2.50 mg)	0	12,7 ± 0,7	0
Mountainous extract (5 mg)	12,65 ± 0,65	15 ± 0,8	0
*OWWE*			
Plain extract (1.25 mg)	0	0	0
Plain extract (2.50 mg)	0	12,7 ± 0,3	0
Plain extract (5 mg)	0	14,55 ± 0,35	0
Mountainous extract (1.25 mg)	0	0	0
Mountainous extract (2.50 mg)	0	0	0
Mountainous extract (5 mg)	11,3 ± 0,3	15,85 ± 0,55	15 ± 0,2
*Amoxicillin (25 μg) *	0	0	15,45 ± 0,45
*Chloramphenicol (30 μg)*	28,75 ± 0,55	14,2 ± 0,5	22,3 ± 0,5
*Ceftriaxone (30 μg)*	19,05 ± 0,45	15,6 ± 0,4	25,8 ± 0,2

Diameter of zone of inhibition (mm) including diameter of 6 mm disc. Results quoted as the average of three readings ± standard deviation. 0 mm indicates no visible zone of inhibition.

## References

[1] Harman D. (1956). Aging: a theory based on free radical and radiation chemistry. *Journal of Gerontology*.

[2] Djeridane A., Yousfi M., Nadjemi B., Boutassouna D., Stocker P., Vidal N. (2006). Antioxidant activity of some Algerian medicinal plants extracts containing phenolic compounds. *Food Chemistry*.

[3] Bertin L., Ferri F., Scoma A., Marchetti L., Fava F. (2011). Recovery of high added value natural polyphenols from actual olive mill wastewater through solid phase extraction. *Chemical Engineering Journal*.

[4] Obied H. K., Bedgood D. R., Prenzler P. D., Robards K. (2008). Effect of processing conditions, prestorage treatment, and storage conditions on the phenol content and antioxidant activity of olive mill waste. *Journal of Agricultural and Food Chemistry*.

[5] Suárez M., Romero M.-P., Ramo T., Macià A., Motilva M.-J. (2009). Methods for preparing phenolic extracts from olive cake for potential application as food antioxidants. *Journal of Agricultural and Food Chemistry*.

[6] Sheabar F. Z., Neeman I. (1988). Separation and concentration of natural antioxidants from the rape of olives. *Journal of the American Oil Chemists' Society*.

[7] Link K. P., Angell H., Walker J. (1929). The isolation of protocatechic acid from pigmented onion scales and its significance in relation to disease resistance in onions. *The Journal of Biological Chemistry*.

[8] Moreno E., Quevedo-Sarmiento J., Ramos-Cormenzana A. (1989). Antibacterial activity of wastewaters from olive oil mills. *Encyclopedia of Environmental Control Technology*.

[9] Capasso R., Evidente A., Schivo L., Orru G., Marcialis M. A., Cristinzio G. (1995). Antibacterial polyphenols from olive oil mill waste waters. *Journal of Applied Bacteriology*.

[10] Niaounakis M., Halvadakis C. P. (2004). Olive-mill waste management. *Literature Review and Patent Survey*.

[11] Bisignano G., Tomaino A., Lo Cascio R., Crisafi G., Uccella N., Saija A. (1999). On the *in vitro* antimicrobial activity of oleuropein and hydroxytyrosol. *Journal of Pharmacy and Pharmacology*.

[12] Aziz N. H., Farag S. E., Mousa L. A. A., Abo-Zaid M. A. (1998). Comparative antibacterial and antifungal effects of some phenolic compounds. *Microbios*.

[13] Alu'datt M. H., Alli I., Ereifej K., Alhamad M., Al-Tawaha A. R., Rababah T. (2010). Optimisation, characterisation and quantification of phenolic compounds in olive cake. *Food Chemistry*.

[14] Obied H. K., Bedgood D. R., Prenzler P. D., Robards K. (2007). Bioscreening of Australian olive mill waste extracts: biophenol content, antioxidant, antimicrobial and molluscicidal activities. *Food and Chemical Toxicology*.

[15] Aranda E., García-Romera I., Ocampo J. A. (2007). Chemical characterization and effects on *Lepidium sativum* of the native and bioremediated components of dry olive mill residue. *Chemosphere*.

[16] Serra A., Rubió L., Borràs X., Macià A., Romero M.-P., Motilva M.-J. (2012). Distribution of olive oil phenolic compounds in rat tissues after administration of a phenolic extract from olive cake. *Molecular Nutrition & Food Research*.

[17] Ramos P., Santos S. A. O., Guerra Â. R. (2013). Valorization of olive mill residues: antioxidant and breast cancer antiproliferative activities of hydroxytyrosol-rich extracts derived from olive oil by-products. *Industrial Crops and Products*.

[18] Romero C., Brenes M., García P., Garrido A. (2002). Hydroxytyrosol 4-*β*-D-glucoside, an important phenolic compound in olive fruits and derived products. *Journal of Agricultural and Food Chemistry*.

[19] Suárez M., Romero M.-P., Motilva M.-J. (2010). Development of a phenol-enriched olive oil with phenolic compounds from olive cake. *Journal of Agricultural and Food Chemistry*.

[20] De La Torre-Carbot K., Jauregui O., Gimeno E., Castellote A. I., Lamuela-Raventós R. M., López-Sabater M. C. (2005). Characterization and quantification of phenolic compounds in olive oils by solid-phase extraction, HPLC-DAD, and HPLC-MS/MS. *Journal of Agricultural and Food Chemistry*.

[21] Alhamad M. N., Rababah T. M., Al-u'datt M. (2012). The physicochemical properties, total phenolic, antioxidant activities, and phenolic profile of fermented olive cake. *Arabian Journal of Chemistry*.

[22] Obied H. K., Allen M. S., Bedgood D. R., Prenzler P. D., Robards K., Stockmann R. (2005). Bioactivity and analysis of biophenols recovered from olive mill waste. *Journal of Agricultural and Food Chemistry*.

[23] Dermeche S., Nadour M., Larroche C., Moulti-Mati F., Michaud P. (2013). Olive mill wastes: biochemical characterizations and valorization strategies. *Process Biochemistry*.

[24] Cardoso S. M., Guyot S., Marnet N., Lopes-da-Silva J. A., Renard C. M. G. C., Coimbra M. A. (2005). Characterisation of phenolic extracts from olive pulp and olive pomace by electrospray mass spectrometry. *Journal of the Science of Food and Agriculture*.

[25] Amro B., Aburjai T., Al-Khalil S. (2002). Antioxidative and radical scavenging effects of olive cake extract. *Fitoterapia*.

[26] Kim K. S., Lee S., Lee Y. S. (2003). Anti-oxidant activities of the extracts from the herbs of *Artemisia apiacea*. *Journal of Ethnopharmacology*.

[27] Lesage-Meessen L., Navarro D., Maunier S. (2001). Simple phenolic content in olive oil residues as a function of extraction systems. *Food Chemistry*.

[28] De Marco E., Savarese M., Paduano A., Sacchi R. (2007). Characterization and fractionation of phenolic compounds extracted from olive oil mill wastewaters. *Food Chemistry*.

[29] Allouche N., Fki I., Sayadi S. (2004). Toward a high yield recovery of antioxidants and purified hydroxytyrosol from olive mill wastewaters. *Journal of Agricultural and Food Chemistry*.

[30] Juárez M. J. B., Zafra-Gómez A., Luzón-Toro B. (2008). Gas chromatographic-mass spectrometric study of the degradation of phenolic compounds in wastewater olive oil by *Azotobacter chroococcum*. *Bioresource Technology*.

[31] Singleton V. L., Rossi J. A. (1965). Colorimetry of total phenolics with phosphomolybdic-phosphotungstic acid reagents. *American Journal of Enology and Viticulture*.

[32] Scalbert A., Monties B., Janin G. (1989). Tannins in wood: comparison of different estimation methods. *Journal of Agricultural and Food Chemistry*.

[33] Kartal N., Sokmen M., Tepe B., Daferera D., Polissiou M., Sokmen A. (2007). Investigation of the antioxidant properties of *Ferula orientalis* L. using a suitable extraction procedure. *Food Chemistry*.

[34] Celiktas O. Y., Kocabas E. E. H., Bedir E., Sukan F. V., Ozek T., Baser K. H. C. (2007). Antimicrobial activities of methanol extracts and essential oils of *Rosmarinus officinalis*, depending on location and seasonal variations. *Food Chemistry*.

[35] Leouifoudi I., Zyad A., Amechrouq A., Oukerrou M. A., Mouse H. A., Mbarki M. (2014). Identification and characterisation of phenolic compounds extracted from Moroccan olive mill wastewater. *Food Science and Technology*.

[36] Rafiq M. S., Rafiq M. I., Khan T., Rafiq M., Khan M. M. (2015). Effectiveness of simple control measures on methicillin resistant *Staphylococcus aureus* infection status and characteristics with susceptibility patterns in a teaching hospital in Peshawar. *Journal of the Pakistan Medicine Association*.

[37] da Costa Andrade V., del Busso Zampieri B., Ballesteros E. R., Pinto A. B., Fernandes Cardoso de Oliveira A. J. (2015). Densities and antimicrobial resistance of *Escherichia coli* isolated from marine waters and beach sands. *Environmental Monitoring and Assessment*.

[38] Do Prado A. C. P., Da Silva H. S., Da Silveira S. M. (2014). Effect of the extraction process on the phenolic compounds profile and the antioxidant and antimicrobial activity of extracts of pecan nut [*Carya illinoinensis* (Wangenh) C. Koch] shell. *Industrial Crops and Products*.

[39] Athamena S., Chalghem I., Kassah-Laouar A., Laroui S., Khebri S. (2010). Activité antioxydante et antimicrobienne d'extraits de Cumain Cyminum L.. *Lebanese Science Journal*.

[40] Fki I., Bouaziz M., Sahnoun Z., Sayadi S. (2005). Hypocholesterolemic effects of phenolic-rich extracts of *Chemlali* olive cultivar in rats fed a cholesterol-rich diet. *Bioorganic and Medicinal Chemistry*.

[41] Yangui T., Sayadi S., Gargoubi A., Dhouib A. (2010). Fungicidal effect of hydroxytyrosol-rich preparations from olive mill wastewater against *Verticillium dahliae*. *Crop Protection*.

[42] Shan B., Cai Y.-Z., Brooks J. D., Corke H. (2007). The *in vitro* antibacterial activity of dietary spice and medicinal herb extracts. *International Journal of Food Microbiology*.

[43] Askun T., Tumen G., Satil F., Ates M. (2009). *In vitro* activity of methanol extracts of plants used as spices against *Mycobacterium tuberculosis* and other bacteria. *Food Chemistry*.

[44] Soler-Rivas C., Espiń J. C., Wichers H. J. (2000). Oleuropein and related compounds. *Journal of the Science of Food and Agriculture*.

[45] Didry N., Seidel V., Dubreuil L., Tillequin F., Bailleul F. (1999). Isolation and antibacterial activity of phenylpropanoid derivatives from *Ballota nigra*. *Journal of Ethnopharmacology*.

[46] Pérez J., Dela Rubia T., Moreno J., Martínez J. (1992). Phenolic content and antibacterial activity of olive oil waste waters. *Environmental Toxicology and Chemistry*.

[47] Hadacek F., Greger H. (2000). Testing of antifungal natural products: methodologies, comparability of results and assay choice. *Phytochemical Analysis*.

[48] Hayouni E. A., Abedrabba M., Bouix M., Hamdi M. (2007). The effects of solvents and extraction method on the phenolic contents and biological activities *in vitro* of Tunisian *Quercus coccifera* L. and *Juniperus phoenicea* L. fruit extracts. *Food Chemistry*.

[49] Guesmi A., Boudabous A. (2006). Activité antimicrobienne de cinq huiles essentielles associées dans les produits de thalassothérapie. *Revue des Régions Arides*.

